# Genome-wide association study of *Mycoplasma anserisalpingitidis* strains for antibiotic susceptibility

**DOI:** 10.1038/s41598-026-39804-w

**Published:** 2026-02-24

**Authors:** Áron B. Kovács, Enikő Wehmann, Katinka Bekő, Dénes Grózner, Krisztina Bali, Zsuzsa Kreizinger, Anna Sawicka, Krisztián Bányai, Miklós Gyuranecz

**Affiliations:** 1 HUN-REN Veterinary Medical Research Institute, Hungária Krt. 21, Budapest, 1143 Hungary; 2 National Laboratory of Infectious Animal Diseases, Antimicrobial Resistance, Veterinary Public Health and Food Chain Safety, Hungária Krt. 21, Budapest, 1143 Hungary; 3 Centre for Metagenomics, University of Debrecen, Debrecen, Hungary; 4 Department of Poultry Diseases, National Veterinary Research Institute, Aleja Partyzantow 57, 24-100 Pulawy, Poland; 5 University of Veterinary Medicine, H-1078 István U. 2, Budapest, Hungary; 6 Molliscience Kft., 2051 Március 15. Utca 1., Biatorbágy, Hungary

**Keywords:** Waterfowl, Whole genome analysis, Antibiotic susceptibility, Macrolide, Lincosamide, Tetracyline, Aminocyclitol, Fluoroquinolone, Pleuromutilin, Antimicrobial resistance, Bacterial infection

## Abstract

*Mycoplasma anserisalpingitidis* is a facultative pathogenic bacterium affecting waterfowl, predominantly geese and sporadically ducks. Understanding the molecular basis of antimicrobial resistance mechanisms is crucial in the preservation of antibiotic efficiency. This study aimed to elucidate the genetic background of antibiotic susceptibility profiles of 110 M*. anserisalpingitidis* strains against nine antimicrobial agents. Significant associations between k-mers and five (tylvalosin, tilmicosin, enrofloxacin, lincomycin, spectinomycin) of the nine antimicrobial agents were identified by pyseer. Significant associations were found in multiple coding sequences that encode various members of efflux pumps, epigenetic regulation and topoisomerases among many other groups of functions. Certain k-mers associated with genes found putative prophage-like sequences suggest potential horizontal gene transfer events that could facilitate the acquisition of novel resistance mechanisms. Based on our findings, the genetic background of antimicrobial resistance of *M. anserisalpingitidis* is composed of multiple factors. Our results not only correlated with the majority of known antibiotic resistance mechanisms (e.g. drug target modification, efflux pumps, methyltransferases) but also showed potentially novel genes that could play a significant role in antimicrobial resistance. The results may serve to expedite the diagnosis of *M. anserisalpingitidis* antibiotic susceptibility profiles and support the fight against the spreading of resistance.

**Supplementary Information:**

The online version contains supplementary material available at 10.1038/s41598-026-39804-w.

## Introduction

*Mycoplasma anserisalpingitidis* is a waterfowl facultative pathogenic bacterium, primarily found in geese and occasionally infecting ducks^1^. Mycoplasmas lack cell wall and are among the smallest known prokaryotes in terms of genome size. *Mycoplasma anserisalpingitidis* was first isolated in 1983 in Hungary^2^ and was fully characterized in 2020 by Volokhov et al.^1^. This pathogen has been frequently detected in Hungarian geese farms^3^, and it is probably present around the whole world^4,5^, as it has been found in geese in Poland, Ukraine and the Russian Federation^6^, China^4^ and Vietnam. *Mycoplasma anserisalpingitidis* can be part of the host’s normal microbiome; however, clinical manifestation usually occurs due to different stress factors, such as cold weather, inappropriate housing conditions, or sexual activity. Clinical signs can include infertility of eggs, increased embryo lethality, phallus and cloaca inflammation, peritonitis, salpingitis, and airsacculitis, leading to high economic losses^7–9^.

There are multiple ways of combating Mycoplasma infections, like prevention by keeping the flock pathogen-free or vaccinating against the bacteria; however, there are currently no commercially available vaccines against *M. anserisalpingitidis*. In case of a disease occurrence, antimicrobial treatment is the primary option to be used against *M. anserisalpingitidis*.

There is a well-documented increase in resistance toward antimicrobial agents in both human and veterinary medicine. This underscores the necessity of rigorous antimicrobial susceptibility testing to expedite targeted antimicrobial therapy. One commonly used method for antimicrobial susceptibility testing in mycoplasmas is the broth microdilution method^10^; however, it might require multiple weeks. Such a prolonged period without treatment can lead to significant economic losses and demands the development of rapid diagnostic tests.

Multiple environmental factors can drive the dissemination of antimicrobial resistance mechanisms, including selective pressure due to antibiotic therapy or horizontal gene transfer between microorganisms. Variable mechanisms of antimicrobial resistance exist also, depending on the antimicrobial agents, like DNA mutations, increased efflux pump activity or methylation^11^. In-depth genomic analyses may enable researchers to find the link between genotype and phenotype regarding antibiotic resistance and support the development of new diagnostic methods for antibiotic susceptibility testing by designing assays targeting these genetic markers. Several studies analyzed the antibiotic susceptibility in *M. anserisalpingitidis* examining single nucleotide polymorphisms (SNPs), biofilm formation and efflux pump mechanisms^12–14^.

One method of contrasting phenotype and genomic markers is to conduct genome-wide association study (GWAS). Genome-wide association studies have been extensively used to dissect the genomic background of many human diseases, like myocardial infarction^15^ and age-related macular degeneration^16^; however, since these early GWA studies, this method has been applied to microbial phenotypes, such as virulence factors or antimicrobial resistance^17–20^. An advantage of this approach is that it cannot only confirm previously described mutations but can also uncover novel mutations, potentially outside the coding DNA sequences (CDSs) of the bacterial genome, which can help in the development of new diagnostic methods. However, these studies require a large number of bacterial genomes, complete and accurate genome sequences for these, and comprehensive phenotypic data.

As GWA studies use various statistical methods (e.g. Fishers exact test, binomial test, fixed effect generalized linear regression); therefore, the number of samples necessary for the experiment to be successful depends on how strongly a certain phenotype in question correlates with the genotype. This number can vary from approximately a hundred to thousands of samples, with more samples allowing the detection of genetic variances with smaller impacts. With advances in whole genome sequencing, it has become ever easier and cheaper to attain reliable whole-genome sequences (WGS) of different bacteria. As mentioned prior it is important for the sequencing to be precise and the coverage of the sequences to be deep and high standard regarding the Phred score (indicates the measure of the base quality) and the depth (describes how often a given nucleotide has been read in the experiment) of the sequencing should be set up. The third important factor in GWAS is phenotypic data, which can range from binary characteristics (such as manifestation of an illness in animals and humans or the ability to metabolize certain molecules) to continuous (like the physical parameters of the organisms or antimicrobial resistance of a microbe). Certain characteristics are relatively easy to note (e.g. host species, physical parameters), while others have to be acquired through various methods (e.g. antimicrobial resistance profile). There are multiple ways of associating genome sequences with phenotypes, the most prominent ones are checking associations between SNPs; k-mers (“k” length short sequences), or unitigs^21^. All of the aforementioned markers have been well demonstrated in different studies^22–27^.

Despite the widespread usage of GWAS in human medicine and their obvious benefits, microbial GWA studies still heavily lag behind examinations conducted on human illnesses. The first GWA studies have been performed on mycoplasmas only recently (2020), all analyzing *M. bovis*^28–30^. In this study, we aimed to dissect the genetic background of antibiotic susceptibility profiles of 110 M*. anserisalpingitidis* strains against nine antimicrobial agents.

## Results

### Antimicrobial susceptibility testing

The quality control strain (ATCC BAA-2147; CP042295) showed consistent results throughout the study. Strains with elevated minimum inhibitory concentration (MIC) values were found in the cases of all tested antibiotics. Among the three macrolides investigated in this study a wide range of MIC values could be observed, with notably high MIC_50_ and MIC_90_ values recorded for tilmicosin (≥ 64 μg/ml for both MIC_50_ and MIC_90_). Tylosin inhibited at 16 μg/ml the 50% of the strains and at ≥ 64 μg/ml the 90% of the strains, while tylvalosin showed the lowest MIC_50_ value (0.5 μg/ml) and MIC_90_ value (2 μg/ml) against the strains. The MIC values for lincomycin clustered around the MIC_50_ value (2 μg/ml), although one isolate yielded high MIC values (> 64 μg/ml). Tetracyclines displayed broad ranges of the MIC values (≤ 0.25 to > 64 μg/ml for oxytetracycline and 0.078 to > 10 μg/ml for doxycycline), with high MIC_50_ (16 and 5 μg/ml, respectively) and MIC_90_ values (64 and 10 μg/ml, respectively). Spectinomycin exhibited a MIC_50_ of 8 μg/ml, with the majority of strains displaying MIC_50_ or higher values. The MIC values for enrofloxacin showed a wide range (1.25 to > 10 μg/ml), with MIC_50_ and a MIC_90_ values above 10 μg/ml. Tiamulin also showed a wide range of MIC values between 0.078 and 2.5 μg/ml. The MIC_50_ value of tiamulin was 0.625 μg/ml, while the MIC_90_ was 2.5 μg/ml. Individual MIC values against each strain are given in Supplementary Table 1, while MIC value graphs are given in Supplementary Fig. 1.

### Whole genome sequencing and assembly

The short reads from previous studies can be found under PRJNA602215, PRJNA650261 and PRJNA602206. While the sequences from this study can be found under PRJNA856806. Based on the whole genome sequencing from the previous studies^3,31–33^, the average number of short reads was 4 753 178 (Minimum: 739 708, Maximum: 30 383 838) with read lengths between 35 and 151 base pairs. The average Phred score was 35.4 (over 99.9% chance of correct base call) (Minimum: 31.9, Maximum: 35.4). The coverage of the genomes was on average 493.9× (Minimum: 51.7×; Maximum 2268.3×). The average length of the genomes after MAUVE alignment was 943 143 base pairs (bps) (Minimum: 862 389 bps, Maximum: 1 097 017 bps).

### Genotype: phenotype association test and promoter mapping

**Fig. 1:**
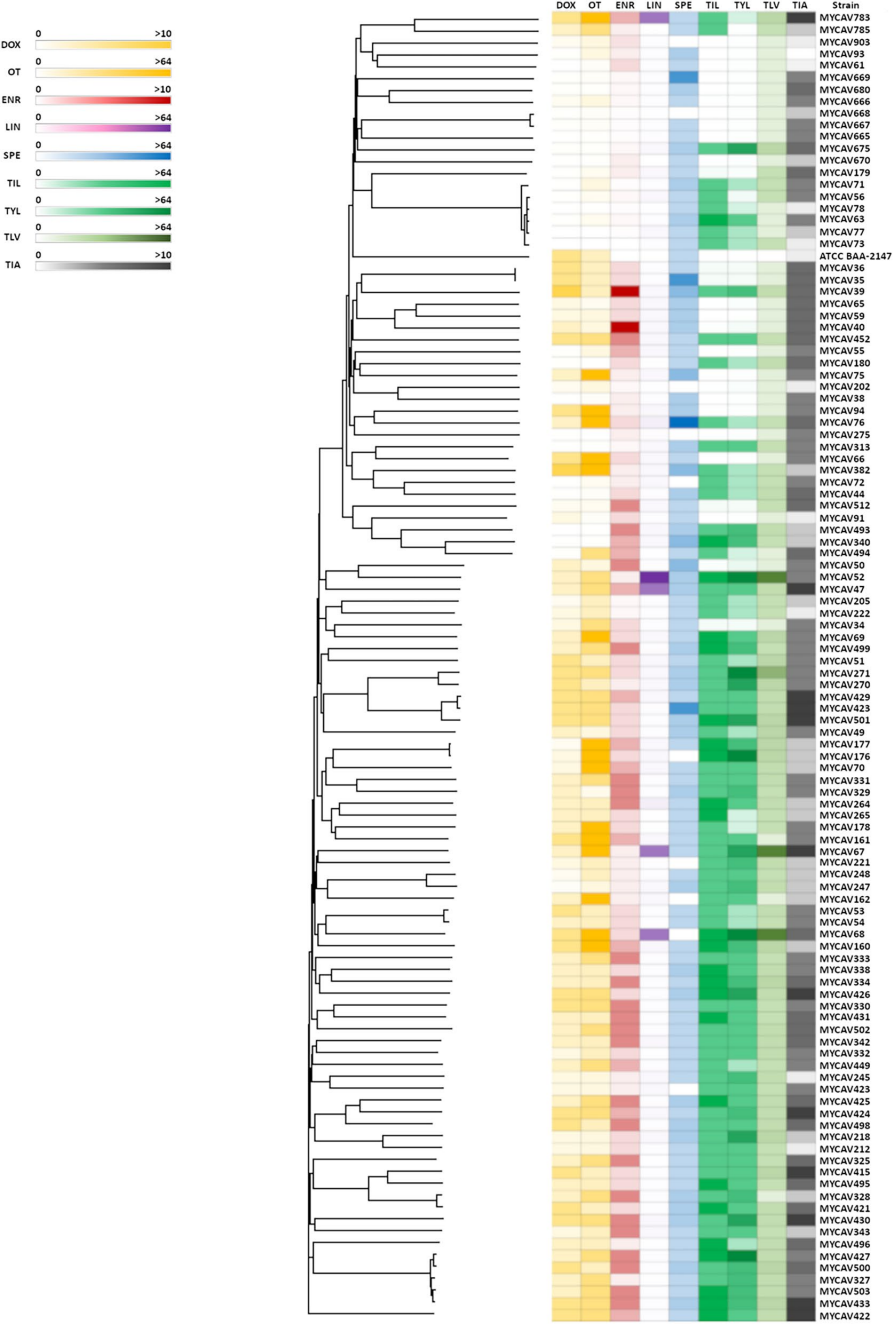
The cgMLST phylogenetic tree of all of the studied *M. anserisalpingitidis* strains along with their antibiotic resistance profile for all of the studied antimicrobial agents. The individual scales for the antimicrobial agents are on the left side of the image (the MIC values are given in μg/ml). Abbreviations: DOX—doxycycline, OT—oxytetracycline, ENR—enrofloxacin, LIN—lincomycin, SPE—spectinomycin, TIL—tilmicosin, TYL—tylosin, TLV—tylvalosin, TIA—tiamulin.

The cgMLST phylogenetic tree and the antimicrobial profiles are presented in Fig. 1. The pyseer tool found various numbers of significant (*p*-value ≤ 5 × 10^–8^) k-mers for 5 of the 9 antimicrobial agents. No k-mer showed significant association with tiamulin resistance neither with tetracycline (oxytetracycline or doxycycline) resistance. For the macrolides no significant k-mer was found for tylosin, but there were significant hits in tylvalosin and tilmicosin (92 and 727 k-mers, respectively). All in all, 19 significant k-mers were found for enrofloxacin, 1 173 significant k-mers for lincomycin and 109 significant k-mers for spectinomycin with pyseer. Promotech found 3 437 promoters in the genome of BAA-2147 type strain.

**Fig. 2:**
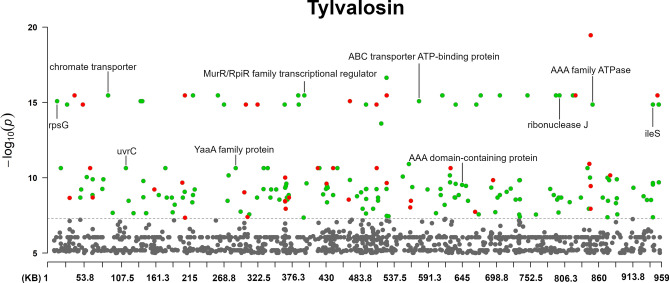
The annotated Manhattan plot of the pyseer GWAS results in case of Tylvalosin. The results are based on the analyses of 105 *Mycoplasma anserisalpingitidis* strains, excluding the atypical *M. anserisalpingitidis* strains. The X axis denotes the position of the k-mers in the type strains (ATCC BAA-2147) genome. The Y axis denotes the − log10 transformation of the *p*-values (e.g. − log_10_(10^–8^) = 8). The annotations are based on the CDS the k-mer was mapped to. Red color denotes CDSs coding hypothetical proteins, while green color shows proteins with known function or domain. The annotations show the CDSs where k-mers with significant associations were mapped. The k-mers were annotated based on protein function and whether or not it can play a role in antibiotic resistance. Certain k-mers have been annotated which may not be present in a protein with known role in antibiotic resistance, nevertheless these CDSs are of interest because of their unusualness or their potential role in the spread of antibiotic resistance (in tylvalosin: chromate transporter, *uvr*C, YaaA family protein, MurP/RpiR family transcriptional regulator, ribonuclease J and *ileS*; in lincomycin: *tsf*, chromate transporter, *uvrC*, SmR/MutS family protein, ZIP family metal transporter, *mnm*E; in spectinomycin: *dna*B, transglutaminase domain-containing protein, *tru*B).

**Fig. 3:**
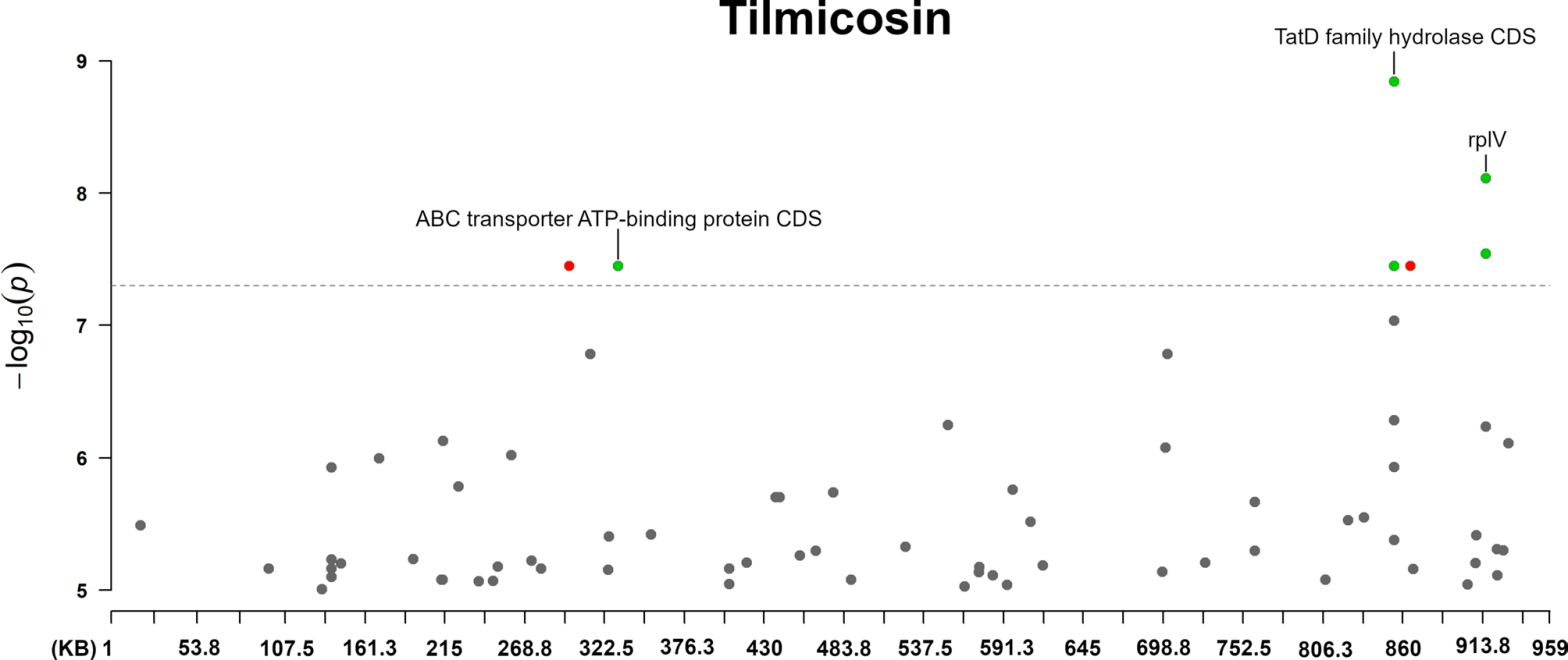
The annotated Manhattan plot of the pyseer GWAS results in case of Tilmicosin. The results are based on the analyses of 105 *Mycoplasma anserisalpingitidis* strains, excluding the atypical *M. anserisalpingitidis* strains. The X axis denotes the position of the k-mers in the type strains (ATCC BAA-2147) genome. The Y axis denotes the − log10 transformation of the *p*-values (e.g. − log_10_(10^–8^) = 8). The annotations are based on the CDS the k-mer was mapped to. Red color denotes CDSs coding hypothetical proteins, while green color shows proteins with known function or domain. The annotations show the CDSs where k-mers with significant associations were mapped. The k-mers were annotated based on protein function and whether or not it can play a role in antibiotic resistance. Certain k-mers have been annotated which may not be present in a protein with known role in antibiotic resistance, nevertheless these CDSs are of interest because of their unusualness or their potential role in the spread of antibiotic resistance (in tylvalosin: chromate transporter, *uvr*C, YaaA family protein, MurP/RpiR family transcriptional regulator, ribonuclease J and *ileS*; in lincomycin: *tsf*, chromate transporter, *uvrC*, SmR/MutS family protein, ZIP family metal transporter, *mnm*E; in spectinomycin: *dna*B, transglutaminase domain-containing protein, *tru*B).

**Fig. 4:**
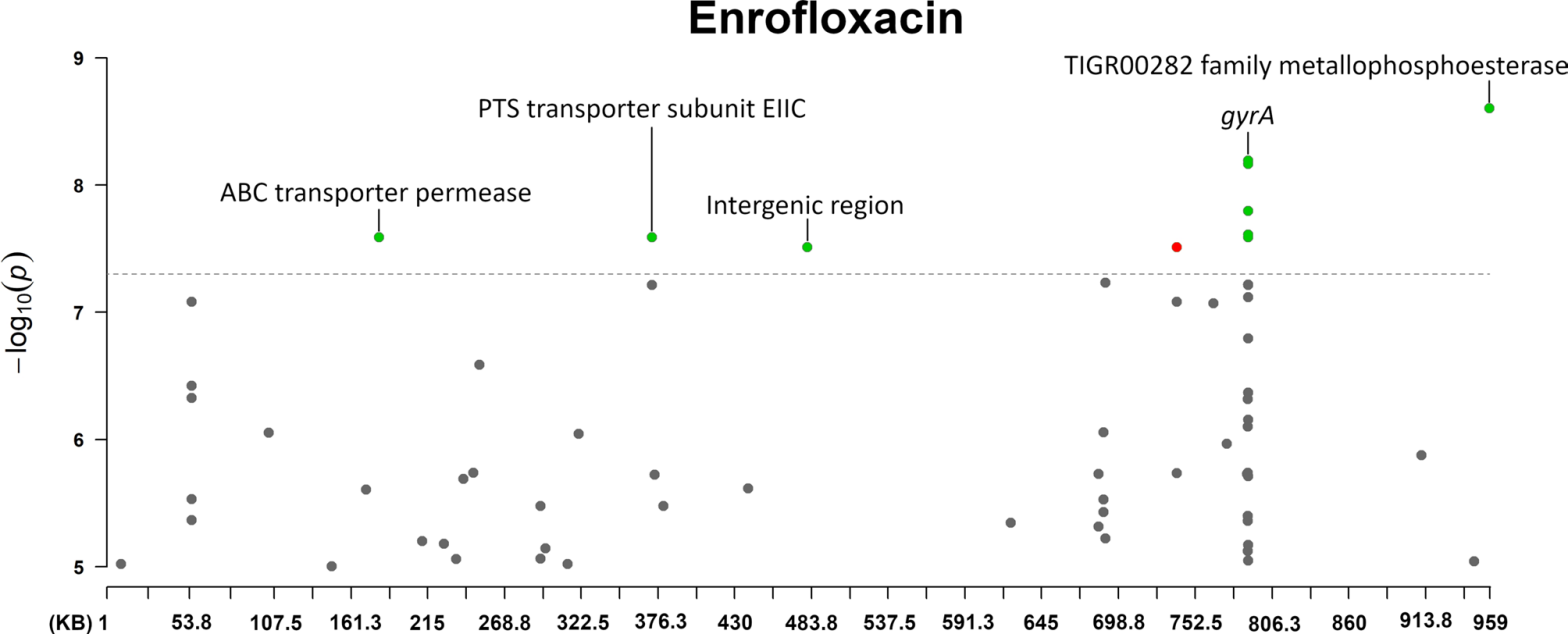
The annotated Manhattan plot of the pyseer GWAS results in case of Enrofloxacin. The results are based on the analyses of 105 *Mycoplasma anserisalpingitidis* strains, excluding the atypical *M. anserisalpingitidis* strains. The X axis denotes the position of the k-mers in the type strains (ATCC BAA-2147) genome. The Y axis denotes the − log10 transformation of the *p*-values (e.g. − log_10_(10^–8^) = 8). The annotations are based on the CDS the k-mer was mapped to. Red color denotes CDSs coding hypothetical proteins, while green color shows proteins with known function or domain. The annotations show the CDSs where k-mers with significant associations were mapped. The k-mers were annotated based on protein function and whether or not it can play a role in antibiotic resistance. Certain k-mers have been annotated which may not be present in a protein with known role in antibiotic resistance, nevertheless these CDSs are of interest because of their unusualness or their potential role in the spread of antibiotic resistance (in tylvalosin: chromate transporter, *uvr*C, YaaA family protein, MurP/RpiR family transcriptional regulator, ribonuclease J and *ileS*; in lincomycin: *tsf*, chromate transporter, *uvrC*, SmR/MutS family protein, ZIP family metal transporter, *mnm*E; in spectinomycin: *dna*B, transglutaminase domain-containing protein, *tru*B).

**Fig. 5:**
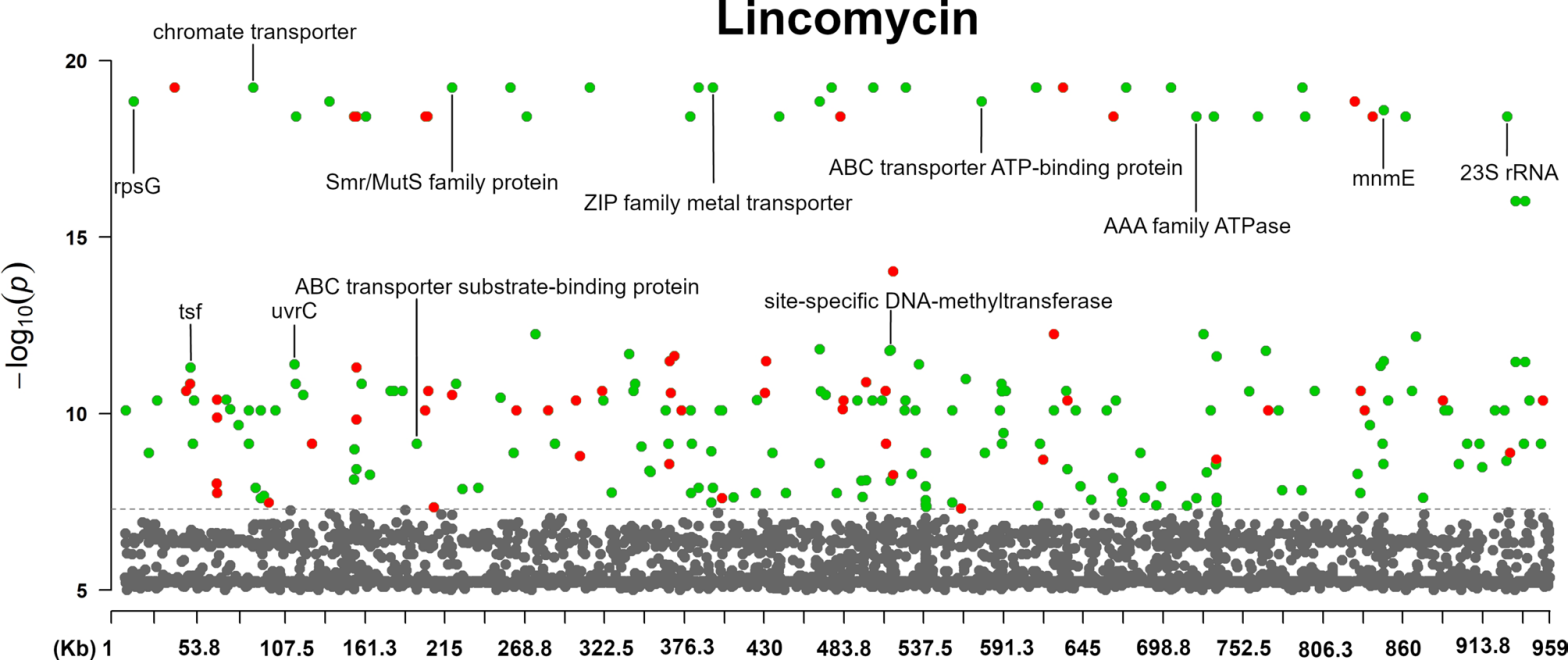
The annotated Manhattan plot of the pyseer GWAS results in case of Lincomycin. The results are based on the analyses of 105 *Mycoplasma anserisalpingitidis* strains, excluding the atypical *M. anserisalpingitidis* strains. The X axis denotes the position of the k-mers in the type strains (ATCC BAA-2147) genome. The Y axis denotes the − log10 transformation of the *p*-values (e.g. − log_10_(10^–8^) = 8). The annotations are based on the CDS the k-mer was mapped to. Red color denotes CDSs coding hypothetical proteins, while green color shows proteins with known function or domain. The annotations show the CDSs where k-mers with significant associations were mapped. The k-mers were annotated based on protein function and whether or not it can play a role in antibiotic resistance. Certain k-mers have been annotated which may not be present in a protein with known role in antibiotic resistance, nevertheless these CDSs are of interest because of their unusualness or their potential role in the spread of antibiotic resistance (in tylvalosin: chromate transporter, *uvr*C, YaaA family protein, MurP/RpiR family transcriptional regulator, ribonuclease J and *ileS*; in lincomycin: *tsf*, chromate transporter, *uvrC*, SmR/MutS family protein, ZIP family metal transporter, *mnm*E; in spectinomycin: *dna*B, transglutaminase domain-containing protein, *tru*B).

**Fig. 6:**
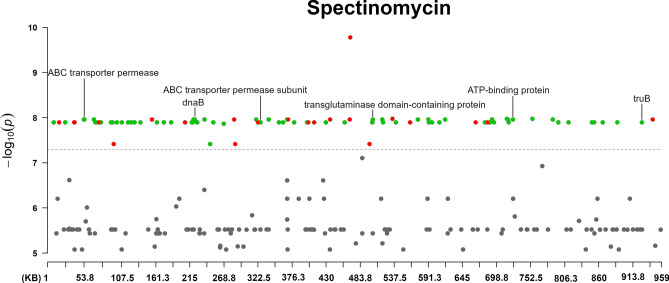
The annotated Manhattan plot of the pyseer GWAS results in case of Spectinomycin. The results are based on the analyses of 105 *Mycoplasma anserisalpingitidis* strains, excluding the atypical *M. anserisalpingitidis* strains. The X axis denotes the position of the k-mers in the type strains (ATCC BAA-2147) genome. The Y axis denotes the − log10 transformation of the *p*-values (e.g. − log_10_(10^–8^) = 8). The annotations are based on the CDS the k-mer was mapped to. Red color denotes CDSs coding hypothetical proteins, while green color shows proteins with known function or domain. The annotations show the CDSs where k-mers with significant associations were mapped. The k-mers were annotated based on protein function and whether or not it can play a role in antibiotic resistance. Certain k-mers have been annotated which may not be present in a protein with known role in antibiotic resistance, nevertheless these CDSs are of interest because of their unusualness or their potential role in the spread of antibiotic resistance (in tylvalosin: chromate transporter, *uvr*C, YaaA family protein, MurP/RpiR family transcriptional regulator, ribonuclease J and *ileS*; in lincomycin: *tsf*, chromate transporter, *uvrC*, SmR/MutS family protein, ZIP family metal transporter, *mnm*E; in spectinomycin: *dna*B, transglutaminase domain-containing protein, *tru*B).

The Manhattan plots of the antibiotic agents are shown in Figs. 2, 3, 4, 5, and 6 (enrofloxacin, lincomycin, spectinomycin, tilmicosin and tylvalosin, respectively).

The most common types of CDS with a significant hit were coding for different transferases (n = 17), especially methyltransferases (n = 7). These were followed by CDSs encoding proteins with efflux pump activity or members of the efflux pump (n = 16). The third most numerous CDSs with positive hits were those coding for ligases and proteins involved in DNA repair (n = 10 in both cases). It is important to note that there were multiple CDSs encoding hypothetical proteins or proteins with unknown or poorly characterized functions. In the case of lincomycin we found significant k-mers in the abortive phage infection protein CDS and the YqaJ viral recombinase family protein CDS, both of which was also found in a previous putative prophage study on *M. anserisalpingitidis*^32^. The function of the CDSs with significant k-mer associations are summarized in Table 1 and detailed in Supplementary dataset 1 (available at 10.6084/m9.figshare.25378600).

**Table 1:**
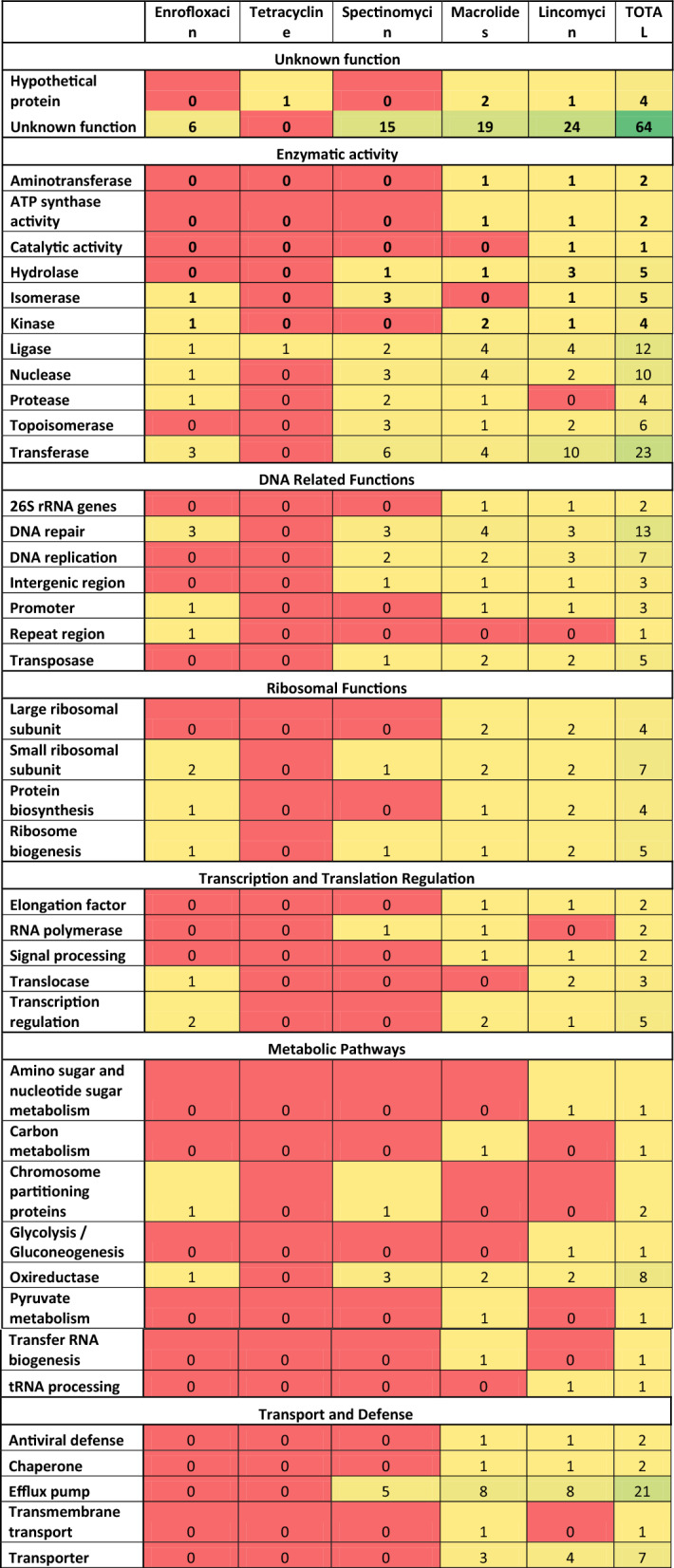
The CDSs with significant k-mer hits.

The CDSs are grouped by functions (rows) and the number of CDSs with significant k-mers in the group are given. The color coding ranges from lowest (red), medium (yellow) to the highest (green) values.

## Discussion

Multiple things can influence the outcome of a genome-wide association study, like the population structure, as the closer the relationship between certain samples or strains the higher the similarities in their genome. Another issue can be the distribution of phenotypic data, if it is skewed too much associating k-mers or SNPs with the phenotype can be problematic. The size of the genome can also influence the necessary sample size of a GWAS, as longer genomes have more genes and thus lead to more statistical comparison and would necessitate a higher sample size. Also, GWAS is only capable of dissecting the genetic background of a trait and unable to account for non-genetic elements of a phenotype, like methylation pattern or small non coding RNAs^34^. These factors can hinder the effectiveness of GWAS, as they are contributing to the phenotype, but not being detectable with DNA sequencing. These can also be the cause behind our study not finding significant k-mers in tiamulin, tetracyclines or in tylosin. As certain resistance associated-genes could be transmitted between pathogens, and bacteria which can infect both animals and humans may develop resistance in the animals and could be transmitted to humans via the food chain, it is extremely important to choose the appropriate agent and dosage in veterinary antimicrobial treatment. Also, some antibiotics are critical in human medicine, while some can only be used in veterinary practice (e.g. enrofloxacin, marbofloxacin, tiamulin and valnemulin) due to toxicity or availability of more potent drugs. It is important to note that mycoplasmas have an intrinsic resistance toward multiple classes of antimicrobial agents, like beta-lactams, glycopeptides and fosfomycin, as these targets the cell wall, which mycoplasmas lack. Mycoplasmas are also resistant to rifampicin due to a mutation in the *rpo*B gene, while polymyxin and sulfonamides/trimethoprim are not effective as mycoplasmas lack lipopolysaccharides and the folic acid pathway, respectively^35^.

Depending on the antimicrobial agent, there are multiple ways in which bacteria can react and defend themselves against it, such as decreasing the uptake of an antimicrobial agent (e.g. against aminoglycosides), altering the target of the antimicrobial agent by mutations in the genes of members of various ribosomal subunits^36^ (e.g. macrolides), or increased efflux pump activity^11^. Pleuromutilins affect bacterial protein synthesis by binding to the 23S rRNA^37^.

Tetracylines hinder the translational process of a bacterial cell by binding to 16S rRNA of the 30S ribosomal subunit and by preventing tRNA binding to 30S at the A site^11^. In the present study significant k-mers in tiamulin, tetracyclines or in tylosin could not be detected, which might be in connection with the above-mentioned difficulties in GWAS, and promotes the search for novel techniques to reveal the mechanisms in these cases. Also, to account for the effect of epigenetic regulation further studies are warranted, with various methods like RNA sequencing or DNA methylation screening.

Macrolides disturb the translation of proteins by targeting the 23S rRNA and resistance mechanisms also overlap with lincosamides. These include methylation of the 23S rRNA through methyltransferases, mutations in the ribosome, through efflux or with macrolide phosphotransferases and esterases. In 2022 our research team^12^ designed mismatch amplification mutation assays to detect nucleotide substitutions potentially associated with decreased susceptibility to macrolides and lincomycin. They analyzed the genes encoding 50S ribosomal proteins and targeted mutations in the 23S rRNA coding genes (*rrl*1 and *rrl*2) along with the 50S ribosomal L22 protein (*rpl*V) coding genes^12^. In 2023 Nagy and co-workers^14^ identified genetic markers in efflux pumps (like ABC-transporter ATP-binding proteins, ABC transporter permease subunits or MATE family efflux transporter) in *M. anserisalpingitidis* mutants with lower susceptibility for the following antimicrobial agents: enrofloxacin, tiamulin, lincomycin, tilmicosin, tylosin, tylvalosin. All of these corresponds with our findings in this study regarding both macrolides (tilmicosin and tylvalosin specifically) and lincomycin. Besides these, in lincomycin we found significant k-mers in other CDSs that match various resistance mechanisms, like methyltransferases and further genes that code members of efflux pumps. Bacterial DNA methylation has been linked to numerous fundamental processes, including transcription, DNA replication, and restriction modification^38^.

Fluoroquinolones (enrofloxacin) inhibit DNA replication by targeting DNA gyrase and topoisomerase IV enzymes, while bacteria can evolve resistance by mutations in the coding genes of these proteins along with efflux of the agents. We found multiple k-mers in CDSs that corresponded with DNA repair (like *recA*, *uvrB* and *ruvA*) or DNA replication (like *dnaN* or *dnaG*), along with various efflux pump component genes.

Aminoglycosides interact with the 30S ribosomal subunit of 16S rRNA causing misreading and truncated proteins and cell death. The resistance mechanism of the bacteria is the modification of aminoglycosides through various enzymes, like acetyltransferases, phosphotransferases, nucleotidyltransferases or 16S ribosomal methylases; however, the mutation of the 16S rRNA gene and increased efflux can also contribute to elevated resistance. We found a diverse set of CDSs with significant k-mers, like a phosphoribosyl transferase, methyltransferases and efflux pump subunits. Interestingly, significant k-mer hits were detected in the DNA gyrase subunit B and DNA topoisomerase IV subunit A CDSs associated with spectinomycin resistance. These two enzyme subunits are not known to be involved in the aminoglycoside resistance, and further studies are required to validate their putative role. In silico methylation screening or RNA sequencing could be employed to further elucidate their functions. Alternatively, repeating GWA study with a larger and more diverse population set of samples might serve to corroborate or refute the current finding.

Our study found that various transferases, especially methyltransferases often show a significant association with altered susceptibility to multiple antimicrobial agents. This corresponds to the fact that various transferases can play key roles in the resistance mechanisms of multiple antibiotics^36^. The second most common type of CDSs with significant hits were either members of efflux pumps or had efflux pump activity. Increase of efflux activity is an efficient way for a bacterium to defend itself against multiple antimicrobial agents. It is often accompanied by a decreased influx of the antibiotic, and these two mechanisms in tandem can lower the antimicrobial agent inside a cell^39^. Significant associations of members of efflux pumps or with efflux pump activity in the present study correspond to the results described earlier, identifying genetic markers in efflux pumps (like ABC-transporter ATP-binding proteins, ABC transporter permease subunits or MATE family efflux transporter) in *M. anserisalpingitidis* mutants with lower susceptibility for certain antimicrobial agents^14^. The two types of CDSs that were the third most common types with significant hits were ligases, many of which are transfer RNA or DNA ligases; and CDSs that code proteins taking part in DNA repair, like recombinases or excinucleases. Furthermore, multiple other types of CDSs previously associated with susceptibility to various antimicrobial agents were detected also in the present study, such as small and large ribosomal subunit protein, 23S rRNA, or gyrases and isomerase genes. However, there were multiple CDSs with significant k-mers, that have not been directly linked with any specific antibiotic resistance mechanisms, but most likely participate in multiple resistance mechanisms. One such group of CDSs was part of the quorum sensing pathway in *M. anserisalpingitidis* based on the KEGG database. Another important finding was the detection of CDSs that have been described in a previous study^32^ as part of prophage-like sequences. This corresponds to the fact that phages can integrate into a bacterial genome under appropriate circumstances and participate in the survival of the bacteria. The presence of multiple hypothetical proteins and uncharacterized CDSs possibly points toward multiple unknown antibiotic resistance pathways. Another hypothesis is that these CDSs are so far non-characterized members of established pathways.

The identification of the k-mers associated with antibiotic susceptibility promotes the development of novel diagnostic tests and support the understanding of resistance mechanisms not only in *M. anserisalpingitidis*, but supposedly in other mycoplasmas and bacteria species. In addition, it is most likely that the combined effects of antibiotic resistance mechanisms maximize the survival of the pathogens.

## Conclusions

While the results of our study correlated with most described antibiotic resistance mechanisms (efflux pumps, methyltransferases, mutations in target genes of antimicrobials), the identification of multiple unidentified or hypothetical protein genes suggests a more intricate genetic background for antimicrobial resistance. The detected genes associated with putative prophage-like sequences suggest potential horizontal gene transfer events that could facilitate the acquisition of novel resistance mechanisms. The genetic background of antimicrobial resistance in the case of *M. anserisalpingitidis* is composed of multiple factors, requiring further investigations to clarify their precise roles.

## Materials and methods

### Antimicrobial susceptibility testing

The antimicrobial susceptibility profiles of 110 M*. anserisalpingitidis* strains have been analyzed in this study. The strains originated from domestic and swan geese and a domestic duck between 1983 and 2019, and were collected from Hungary (n = 99), Poland (n = 8), China (n = 2), and Vietnam (n = 1), from the cloaca (n = 59), phallus and phallus lymph (n = 36), trachea (n = 5), follicle (n = 5), oviduct (n = 1), semen (n = 3) or the lung and air sac (n = 1) of the animals. The metadata of the strains can be found in Supplementary Table 1. According to the written declaration (reference number: VMRI/2018/0024) of the Ethics Committee of the HUN-REN Veterinary Medical Research Institute ethical approval was not required for the study as the samples were taken during routine diagnostic examinations with the written consent of the owner.

The isolation and cell propagation necessary for the antimicrobial susceptibility testing were performed as described in previous studies^12,31,33,40^. In brief, the isolation and propagation were accomplished in Oxoid Mycoplasma broth medium (pH 7.8) (Thermo Fisher Scientific, Inc., Waltham, MA, USA) supplemented with 0.5% (wt/vol) sodium pyruvate, 0.5% (wt/vol) glucose, 0.15% l-arginine hydrochloride and 0.005% (wt/vol) phenol red, incubating the cultures at 37 °C for 1–2 days. Nine antimicrobial agents were tested using the broth microdilution method: a pleuromutilin (tiamulin); two tetracyclines (oxytetracycline and doxycycline); three macrolides (tylosin, tylvalosin, tilmicosin); a lincosamide (lincomycin); an aminocyclitol (spectinomycin) and a fluoroquinolone (enrofloxacin). The dilution and storage of the antibiotics, broth microdilution examinations, and determination of the MIC values were performed as established previously^10^.

In this study the MIC values were arranged in an orderly array and the middle value (0.5 × n (the number of strains—110 in this study)) was selected for MIC_50_. The MIC_90_ value was calculated similarly; however, in this case the 99^th^ value was selected (0.9 × n). The antibiotics were applied two separate ranges, for tylosin, tilmicosin, tylvalosin, lincomycin, oxytetracycline, spectinomycin of 0.25–64 μg/mL, while for doxycycline, enrofloxacin and tiamulin the range was 0.039–10 μg/mL. The MIC values that exceeded the range of the assay were included in the study with a 1.5 multiplier, denoting the fact these values were higher than the range, but could not be determined exactly. Out of the 110 M*. anserisalpingitidis* strains, the MIC values for 31 isolates were previously determined, whereas susceptibility testing of 79 isolates was either performed or redone during this study using the same method^12,40^. The *M. anserisalpingitidis* ATCC BAA-2147 type strain was tested on each microtiter plate to confirm the validity of the results.

### Whole-genome sequencing, assembly, and annotation

The *M. anserisalpingitidis* whole-genome sequences originated from previous studies^31,32^. To summarize, the DNA was extracted from 10 ml of logarithmic-phase broth cultures of the strains using QIAamp DNA Mini kit (Qiagen Inc., Hilden, Germany) following the manufacturer’s instructions. Next-generation sequencing was performed on NextSeq 500 Illumina equipment (Illumina Inc., San Diego, CA, USA), with NextSeq 500/550 High Output Kit v2.5 reagent kit (Illumina Inc.).

The quality check of the short reads was performed using FastQC software version 0.11.9^41^ the quality threshold was a Phred score of 30. A draft genome was assembled for all of the strains with SPAdes version 3.13.1^42^. The contigs of the draft genomes were ordered using ATCC BAA-2147 with the MAUVE algorithm version 20150226^43^ and annotated with the NCBI Prokaryote Genome Annotation Pipeline (PGAP) version 6.2^44^.

### Genotype: phenotype association test and promoter mapping

The preparatory steps necessary for the study, the estimation of genomic distances and counting k-mers were performed based on the pyseer documentation^45^. The core genome multi-locus sequence typing (cgMLST) schema from previous studies^19,27^ was used in this study. The genetic distance of the strains was calculated from the cgMLST schema with the script provided by the pyseer software in order to correct for the population structure. In the following step, the k-mers were counted using fsm-lite software version 1.0^46^, as per the best practice in the pyseer documentation. The number dimensions to retain was calculated using the output of a scree plot python script.

The genome wide association study was conducted with the pyseer software version 1.3.11^47^, following the tutorial and best practices on the pyseer software’s documentation. In short, we used three separate files: MIC values of the samples, the distance matrix from the cgMLST schema, and the k-mers counted by fsm-lite in the previous step. As there are no official cutoff for antibiotic susceptibility in the case of *M. anserisalpingitidis*, the MIC values were treated as continuous variable. Linear mixed model was used in the study to better account for the relatedness of the strains. The association study was repeated for each antimicrobial agent, nine times in total. Due to multiple statistical testing, a more stringent *p*-value criterion is used in genome wide studies, usually *p* < 5 × 10^–8^. Statistically significant k-mers were mapped to the type strain (ATCC BAA-2147; GenBank Accession number: CP042295)^33^. Promotech version 1.0^48^ software was used to search for promoter regions in four strains with complete whole genomes (ATCC BAA-2147; MYCAV93, GenBank Accession number: CP041663; MYCAV177, GenBank Accession number: CP041664 and MYCAV271, GenBank Accession number: CP083178). The promoter regions with a score of 0.8 (as there are only few promoters described in *Mycoplasmas*, this stricter than usual criteria was chosen) have been used to annotate the type strain in Geneious Prime 2023.1.1^49^. The significant k-mers and promoter regions were cross-referenced. After running the association study significant background noise could be observed in the Manhattan plots, as such the five atypical strains (MYCAV61, MYCAV93, MYCAV783, MYCAV785 and MYCAV903), that showed significant divergence in genomic composition, were omitted and the whole procedure was run again, with new distance matrix, k-mers and phenotype files.

## Supplementary Information

Supplementary Information 1.

Supplementary Information 2.

Supplementary Information 3.

## Data Availability

The short reads can be accessed in the NCBI SRA database: PRJNA602215 (https://www.ncbi.nlm.nih.gov/bioproject/?term=PRJNA602215); PRJNA856806 (https://www.ncbi.nlm.nih.gov/bioproject/?term=PRJNA856806); PRJNA650261 (https://www.ncbi.nlm.nih.gov/bioproject/?term=PRJNA650261); PRJNA602206 (https://www.ncbi.nlm.nih.gov/bioproject/?term=PRJNA602206). The draft genome assemblies and the CDSs with significant k-mer hit can be accessed at: under: https://figshare.com/articles/dataset/_i_Mycoplasma_anserisalpingitidis_i_genome_wide_association_study_data/25378600.
