# Supplementary material for: Genome-wide association study of Mycoplasma anserisalpingitidis strains for antibiotic susceptibility
Source: Sci Rep. 2026 Feb 24;16:10306. doi: 10.1038/s41598-026-39804-w (PMC13031706; doi:10.1038/s41598-026-39804-w)
Supplement: Supplementary file 1 — Supplementary Information 1. [file 41598_2026_39804_MOESM1_ESM.docx]

**Supplementary table S1. Background information of the *Mycoplasma anserisalpingitidis* strains**. The table contains the metadata of the strains the MIC values of the strains can be found. Abbreviations: Na – data not available, bp – basepairs; GC% - guanine-cytosine percentage of the genomes. The accession numbers in the table are the NCBI SRA accession numbers.

**Supplementary figure 1. Bar graphs of the MIC values of the antibiotic agents**. The bar graphs of the antimicrobial agents. The X axis denotes the MIC values, while the Y axis denotes the number of strains that were inhibited at that specific concentration.

**Supplementary data**: The draft genomes and the CDSs with significant k-mer hit have been uploaded to the Figshare site (see Availability of data and materials)
