# Supplementary figures and images for: Genome-wide association study of Mycoplasma anserisalpingitidis strains for antibiotic susceptibility
Source: Sci Rep. 2026 Feb 24;16:10306. doi: 10.1038/s41598-026-39804-w (PMC13031706; doi:10.1038/s41598-026-39804-w)

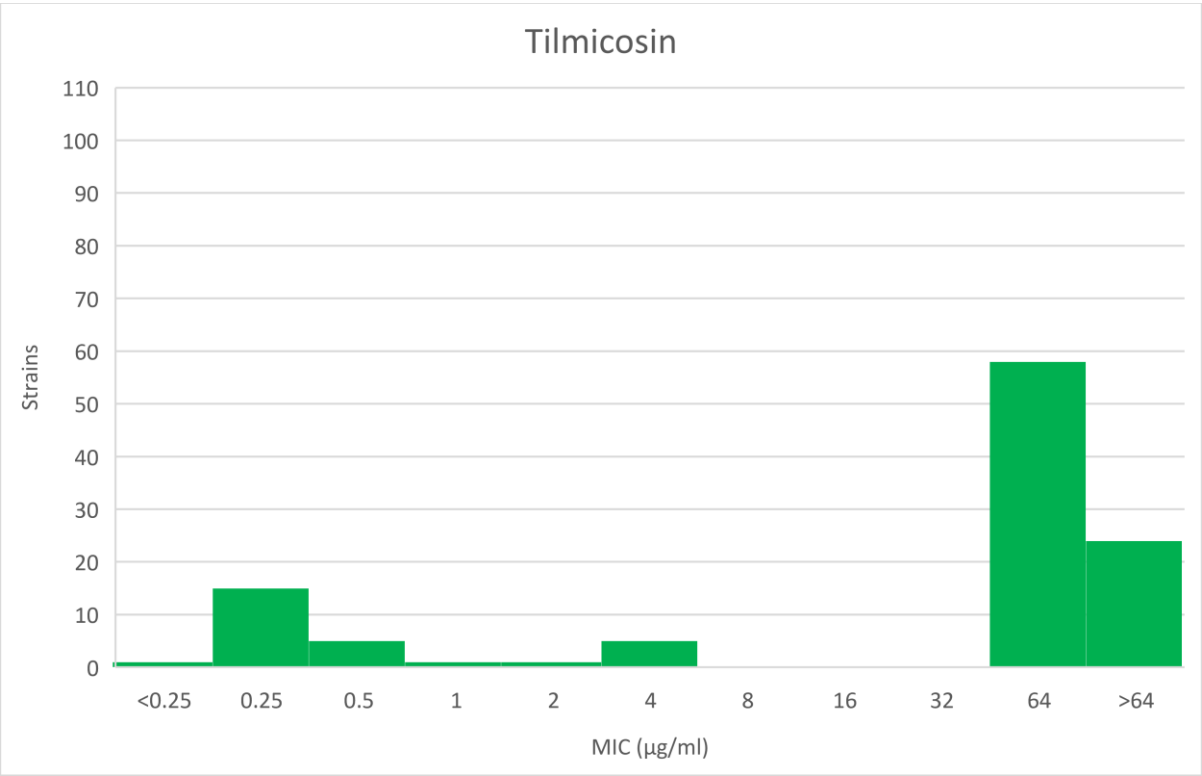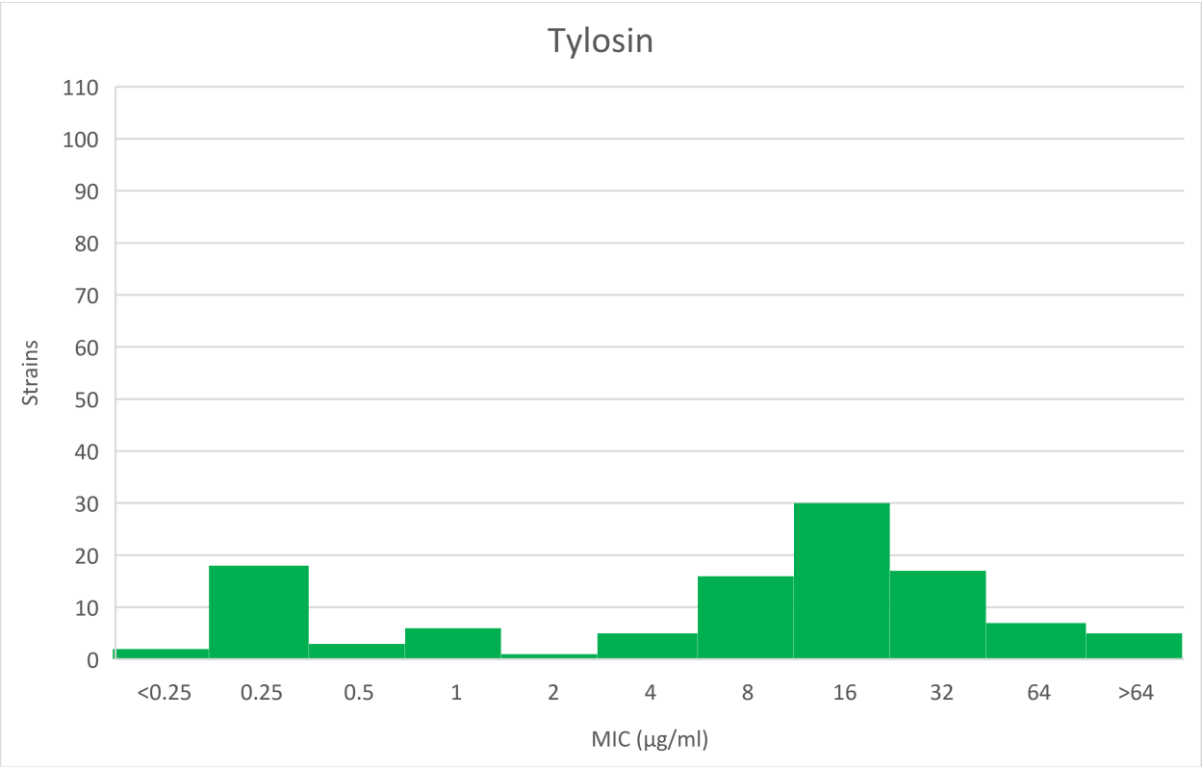

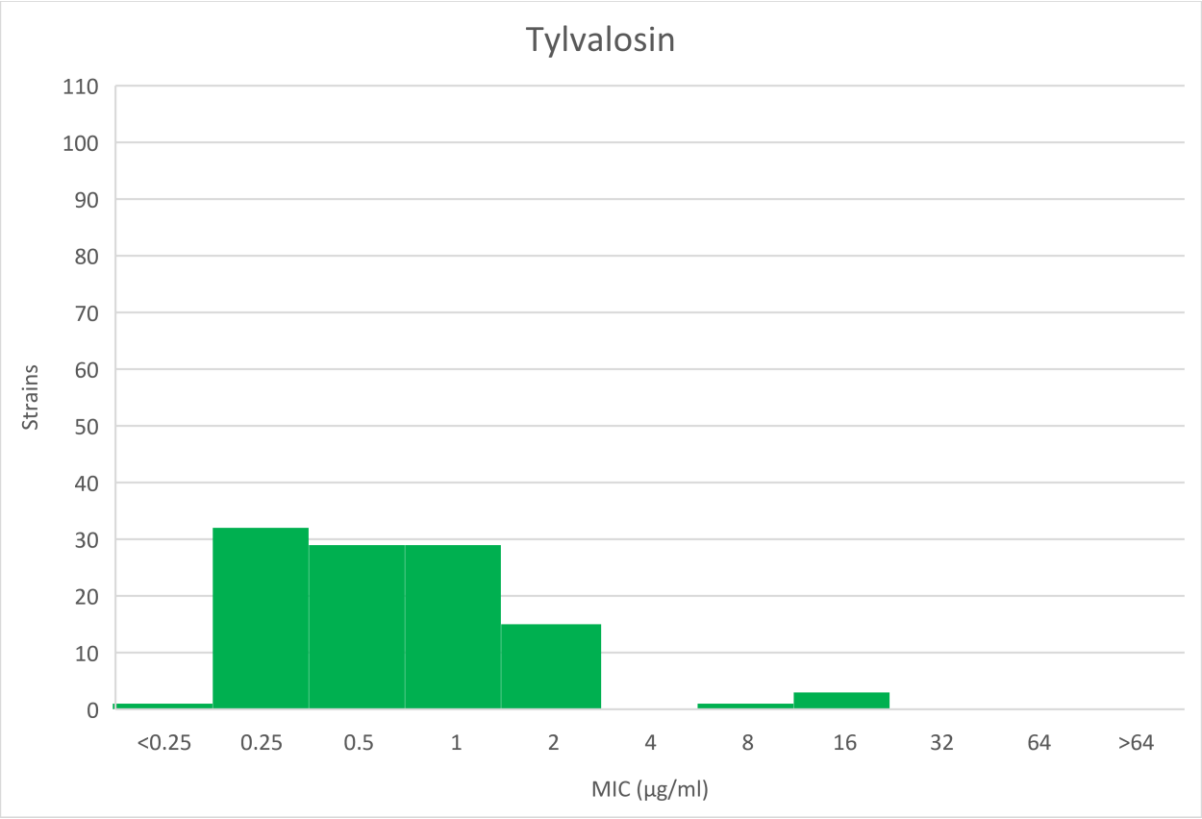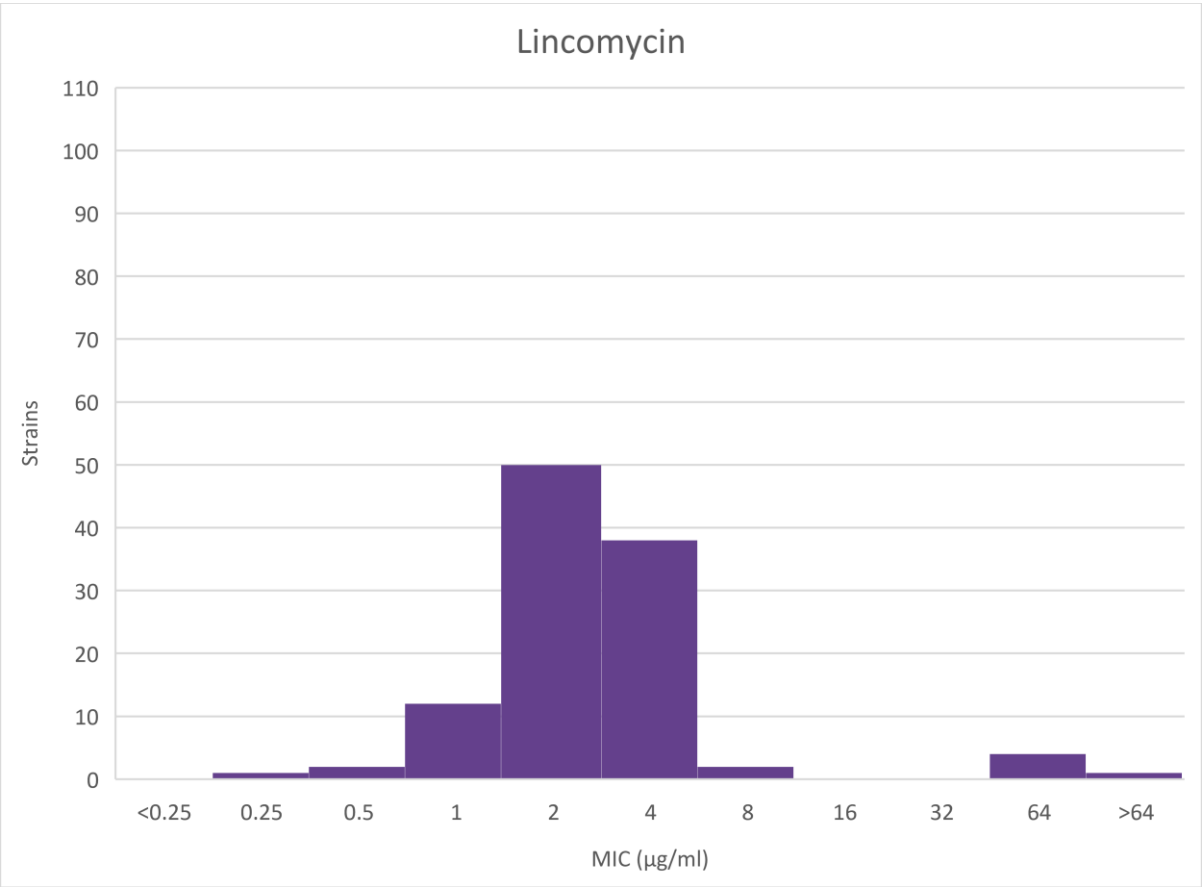

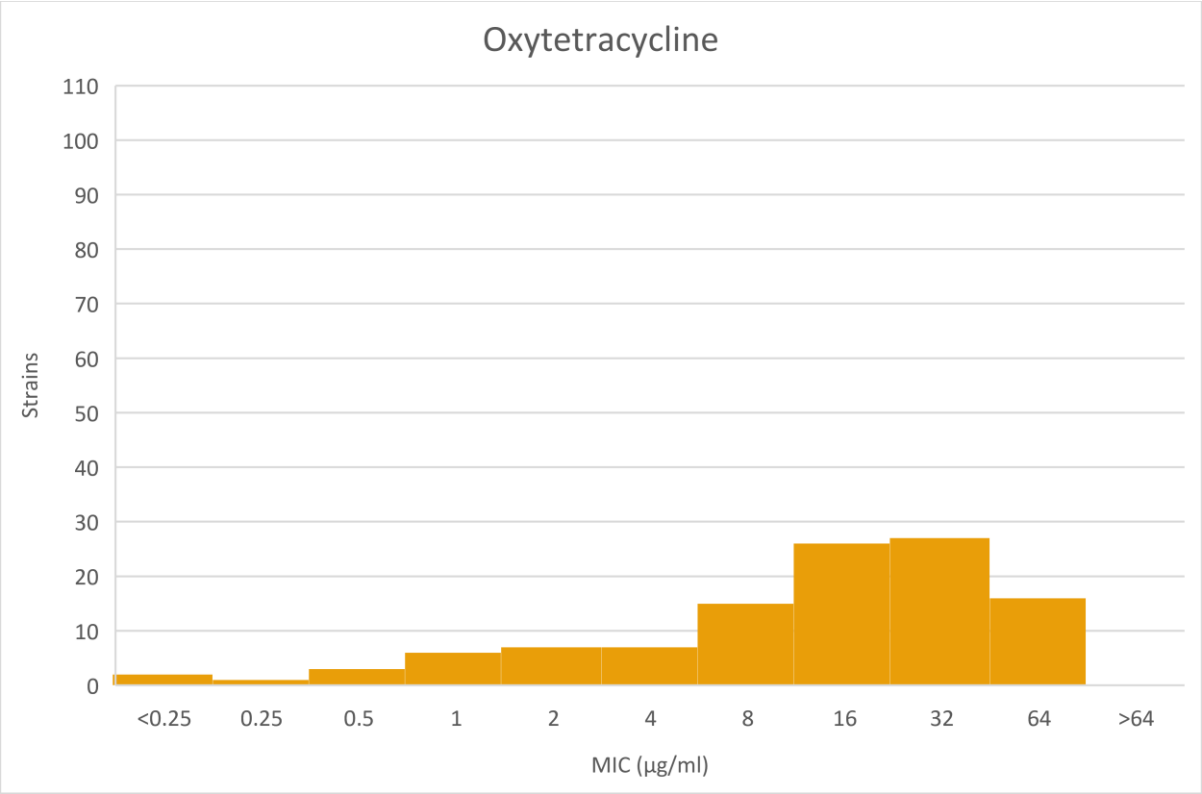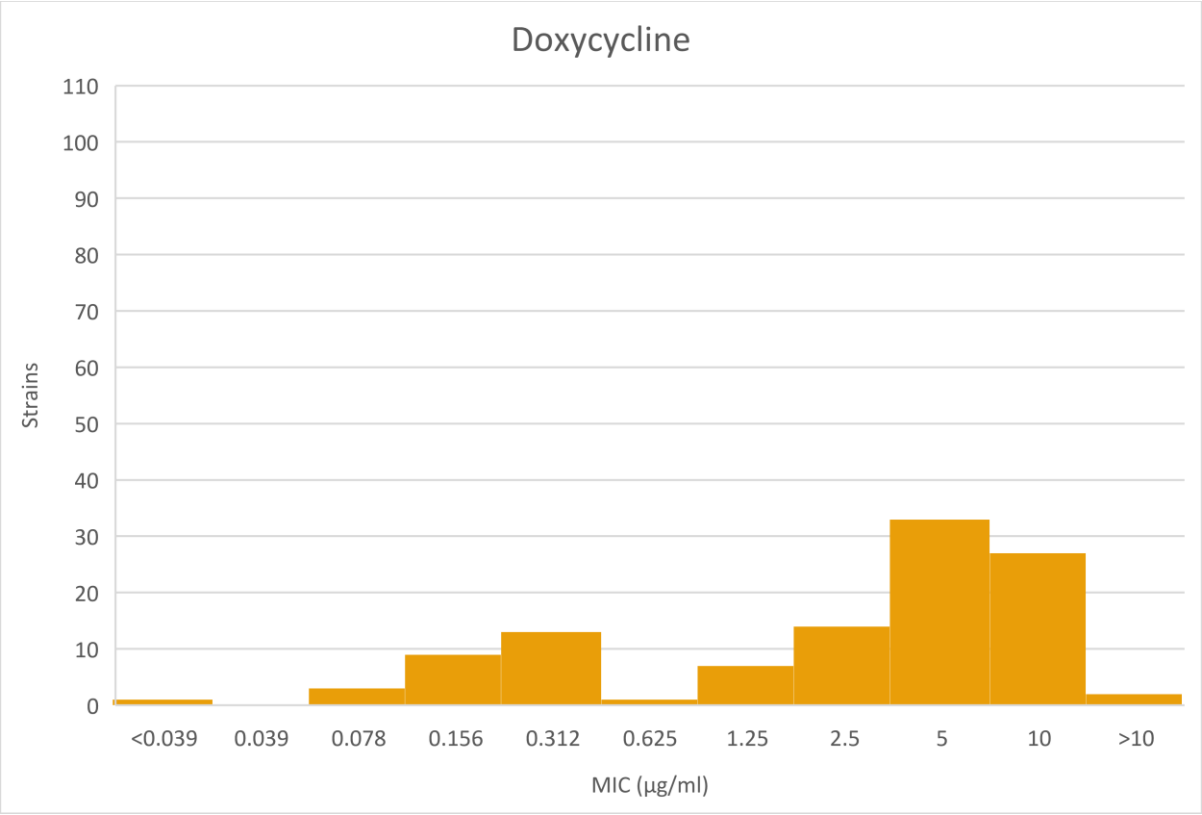

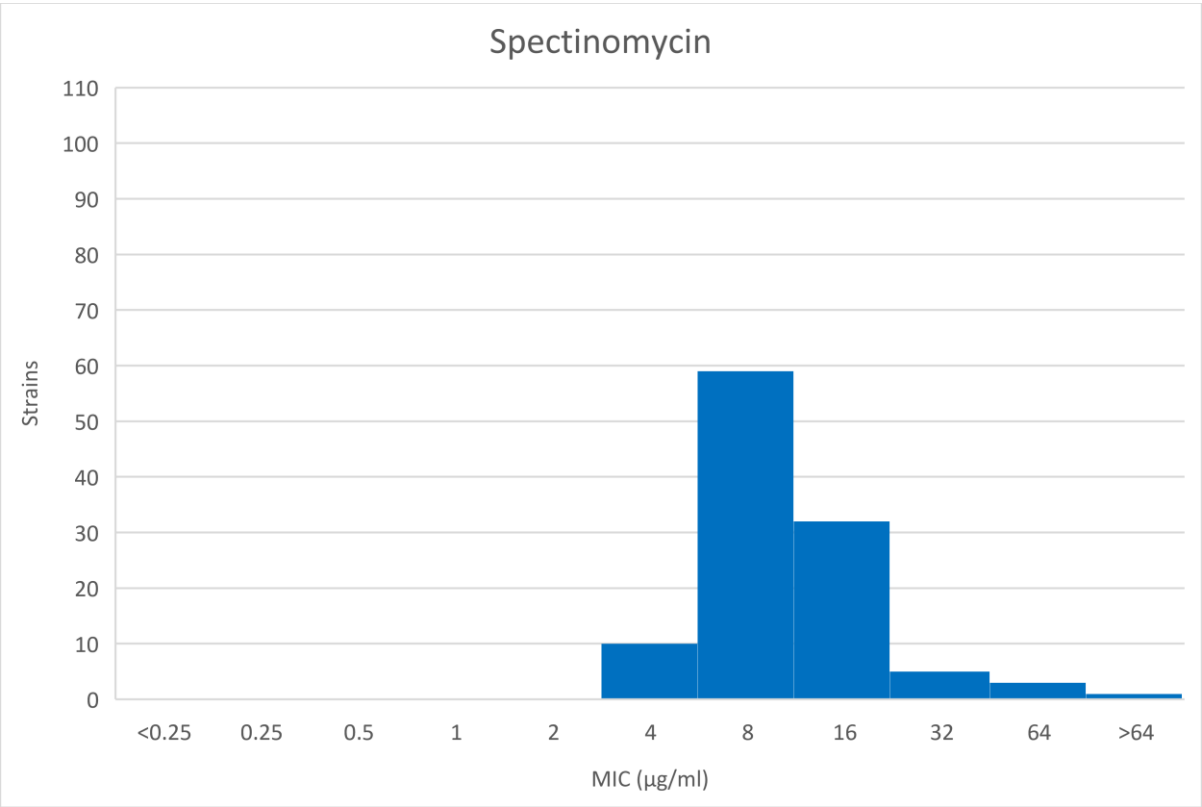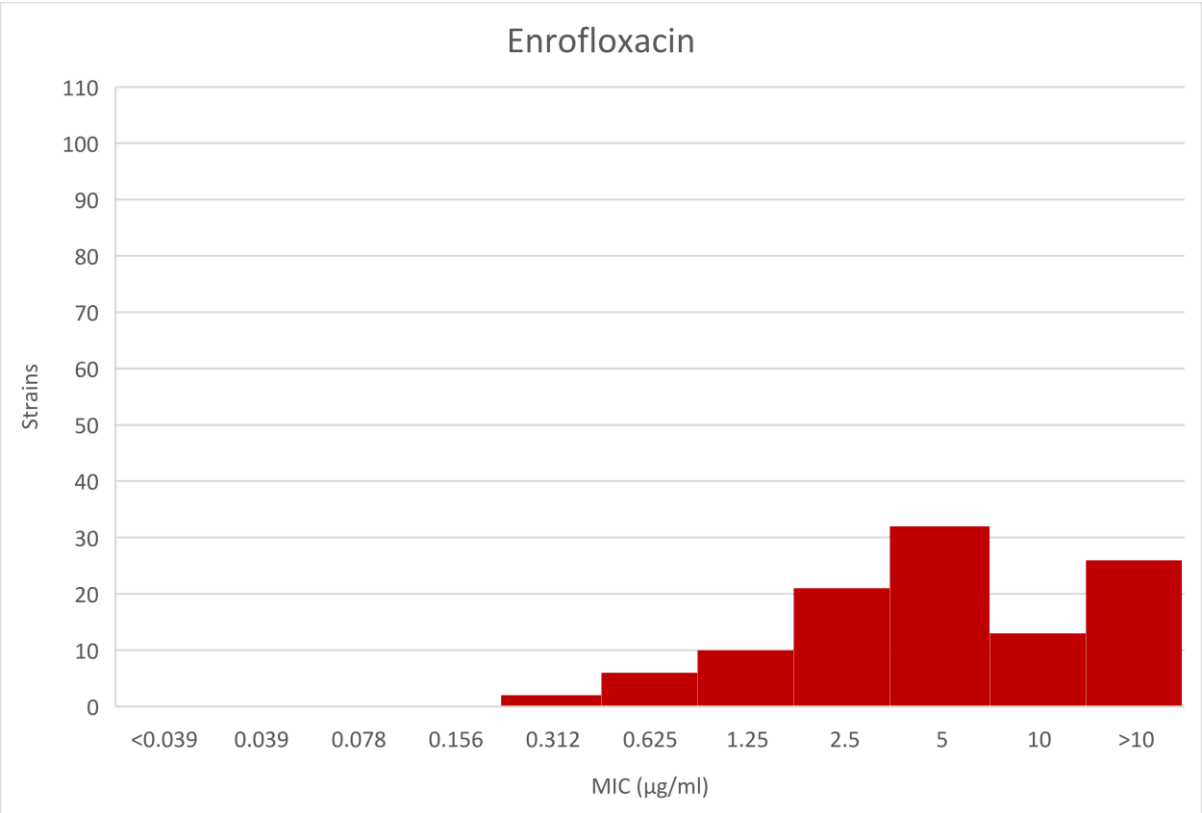

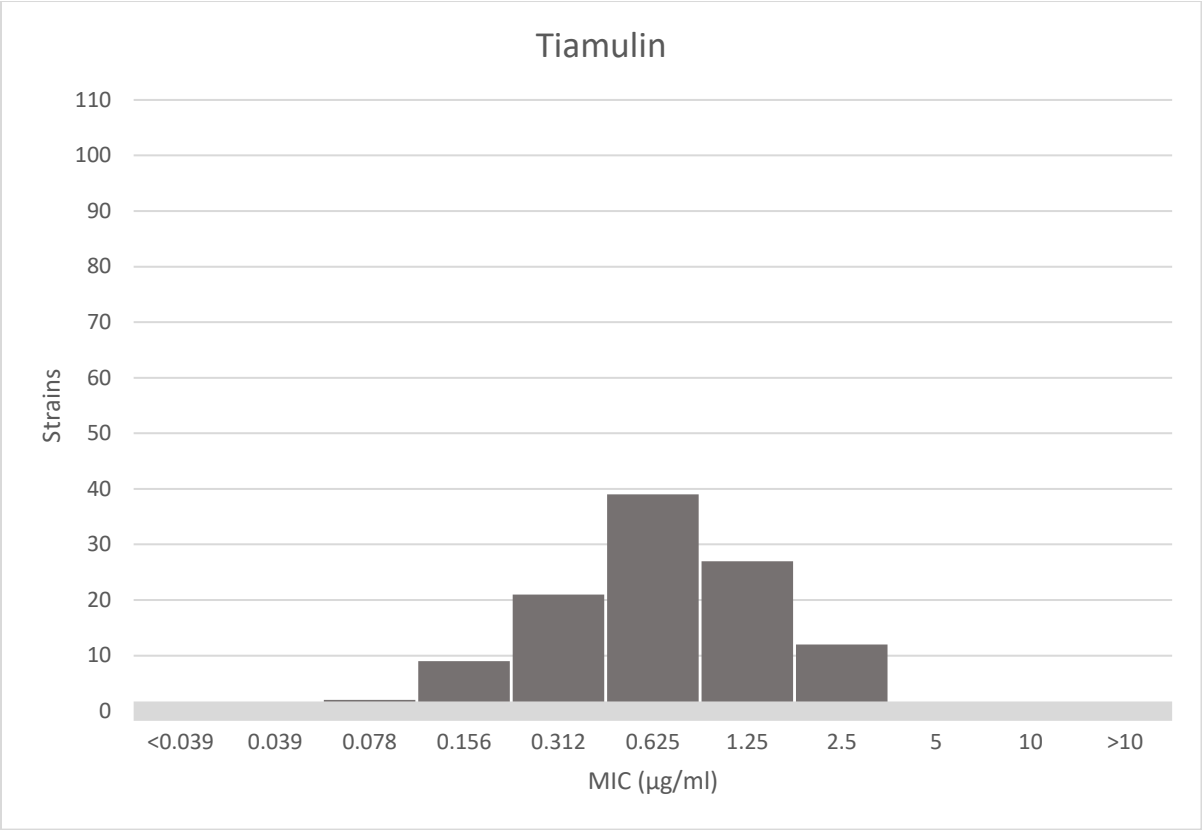

Supplement: Supplementary file 2 — Supplementary Information 2. [file 41598_2026_39804_MOESM2_ESM.pdf]
